# The origin of β-strand bending in globular proteins

**DOI:** 10.1186/s12900-015-0048-y

**Published:** 2015-10-22

**Authors:** Kazuo Fujiwara, Shinichi Ebisawa, Yuka Watanabe, Hiromi Fujiwara, Masamichi Ikeguchi

**Affiliations:** Department of Bioinformatics, Soka University, 1-236 Tangi-cho, Hachioji, Tokyo 192-8577 Japan

**Keywords:** Statistical analysis, Protein design, Hydrophobic cluster, β-strand twist

## Abstract

**Background:**

Many β-strands are not flat but bend and/or twist. However, although almost all β-strands have a twist, not all have a bend, suggesting that the underlying force(s) driving β-strand bending is distinct from that for the twist. We, therefore, investigated the physical origin(s) of β-strand bends.

**Methods:**

We calculated rotation, twist and bend angles for a four-residue short frame. Fixed-length fragments consisting of six residues found in three consecutive short frames were used to evaluate the twist and bend angles of full-length β-strands.

**Results:**

We calculated and statistically analyzed the twist and bend angles of β-strands found in globular proteins with known three-dimensional structures. The results show that full-length β-strand bend angles are related to the nearby aromatic residue content, whereas local bend angles are related to the nearby aliphatic residue content. Furthermore, it appears that β-strands bend to maximize their hydrophobic contacts with an abutting hydrophobic surface or to form a hydrophobic side-chain cluster when an abutting hydrophobic surface is absent.

**Conclusions:**

We conclude that the dominant driving force for full-length β-strand bends is the hydrophobic interaction involving aromatic residues, whereas that for local β-strand bends is the hydrophobic interaction involving aliphatic residues.

**Electronic supplementary material:**

The online version of this article (doi:10.1186/s12900-015-0048-y) contains supplementary material, which is available to authorized users.

## Background

**Fig. 1:**
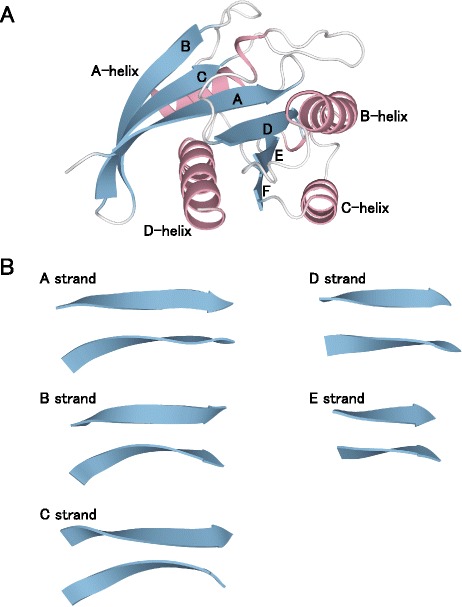
β-strand twisting and bending in Rheb. The small GTPase Rheb (Ras homolog enriched in brain) of the P-loop–containing nucleoside triphosphate hydrolase fold (PDB ID: 1XTQ). **a** Ribbon diagram showing the secondary and tertiary structures of Rheb. The individual β-strands of its twisted sheet are labeled A-E, and its α-helices are labeled A-D. **b** Each of the five β-strands is shown in two different orientations

Many β-strands have a right-hand twist and a bend, which have been suggested to induce a twist in the corresponding β-sheet [1–3]. For example, the small GTPase Ras homolog enriched in brain (Rheb)—which belongs to the P-loop–containing nucleoside triphosphate hydrolase fold as defined in the Structural Classification of Proteins database (SCOP [4])—has a large twisted β-sheet surrounded by four α-helices (Fig. 1). Its first three β-strands are highly bent.

In the early 1980s, simple energy minimization calculations were used to attribute the preference for a right-handed twist to intra-strand and inter-strand nonbonded side-chain interactions [5–7]. Specifically, these studies suggested that, for Ala and Val β-sheets, the major driving force that favors a right-handed twist could be attributed to intra-strand interactions. Wang et al. used molecular dynamics simulations to confirm the tendency of isolated β-strands to assume a twisted conformation, although they reported a very small difference in free energy between twisted and nontwisted conformations of a single strand; this suggested that the twist must also be stabilized by inter-strand interactions [8]. Using a calculation based on density functional theory, Shamovsky et al. showed that the right-handed twisting of β-strands in proteins is an inherent property of the peptide backbone of individual β-strands and that twisting is enhanced by inter-strand hydrogen bonding in multi-stranded β-sheets [9]. Rossmeisl et al. showed that hydrogen-bond strength in a given β-sheet increases with the number of strands in the sheet [10]. Statistical analyses by Ho et al. showed that the intra-strand O⋯Cβ steric clash constrains the range of psi angles (ψ < 116°), resulting in a bias towards right-handed twisting of β-strands [11]. Furthermore, Koh et al. proposed that the β-sheet surface structure is mainly determined by the conformation of β-strand backbones and that the side chains make only small contributions to the surface structure [12]. Thus, these studies indicate that β-sheet structure is mainly determined by the polypeptide backbone framework consisting of backbone atoms and β-carbons.

Furthermore, we recently showed that the most frequent twist angles defined for sequences of four residues (short frames) negatively correlate with the proportion of Ser, Thr, and Asn residues found in the frames [13]. Almost all Ser, Thr, and Asn side-chain oxygen atoms in β-strands contact main-chain nitrogens, which are involved in inter-strand hydrogen bonding. We concluded that these side-chains influence the inter-strand hydrogen bonds, thereby suppressing β-strand twisting.

Thus, although certain interactions that can influence the extent to which a β-strand is twisted have been uncovered, those that influence β-strand bending remain to be elucidated. For our previous report, we characterized only the local right or left (RL) bend angles between adjacent residues in β-strands (see below for a definition of the RL bend angle) and found that these angles strongly correlate with the local twist angles and the number of hydrophobic residues in the examined β-strand [13]. However, there are also twisted β-strands that are not bent, e.g., strands D and E in Fig. 1, suggesting that the overall bend of a β-strand may be independent of its local RL bends and twists.

In this study, we examined how hydrophobic residues might affect local bends and impact full-length β-strand bends. We calculated the direction—up or down (UD)—of local bend angles and also the twist and bend angles of longer β-strand fragments. We found that the bends of the fragments are oriented in the UD direction so as to accommodate nearby hydrophobic residues; these residues, however, do not affect the RL orientation. Furthermore, we found little correlation between the bend and twist angles for six-residue fragments, indicating that the bend angles in these fragments are independent of their twist angles.

## Methods

### Determining amino acid propensities for β-strands

The propensity, P, of each amino acid, to be found in a β-strand was calculated as follows:

1 $$ \mathrm{Pi}=\frac{{{\mathrm{f}}_{\mathrm{i}}}^{\mathrm{B}}}{{\mathrm{f}}_{\mathrm{i}}} $$

where f β_i_ is the frequency of the amino acid i occurring in β-strands, and f_i_ is the frequency of the amino acid i occurring in proteins. We used f_i_ as reported by MaCaldon and Argos [14] for 1021 unrelated proteins. If P_i_ = 1, the amino acid i is contained in the β-strand at the same frequency as it appears in the protein database. If P_i_ > 1, the amino acid i appears more frequently in the β-strand than in the protein database.

### Definitions of twist angles, rotation angles and UD and RL bend angles for a short frame

**Fig. 2:**
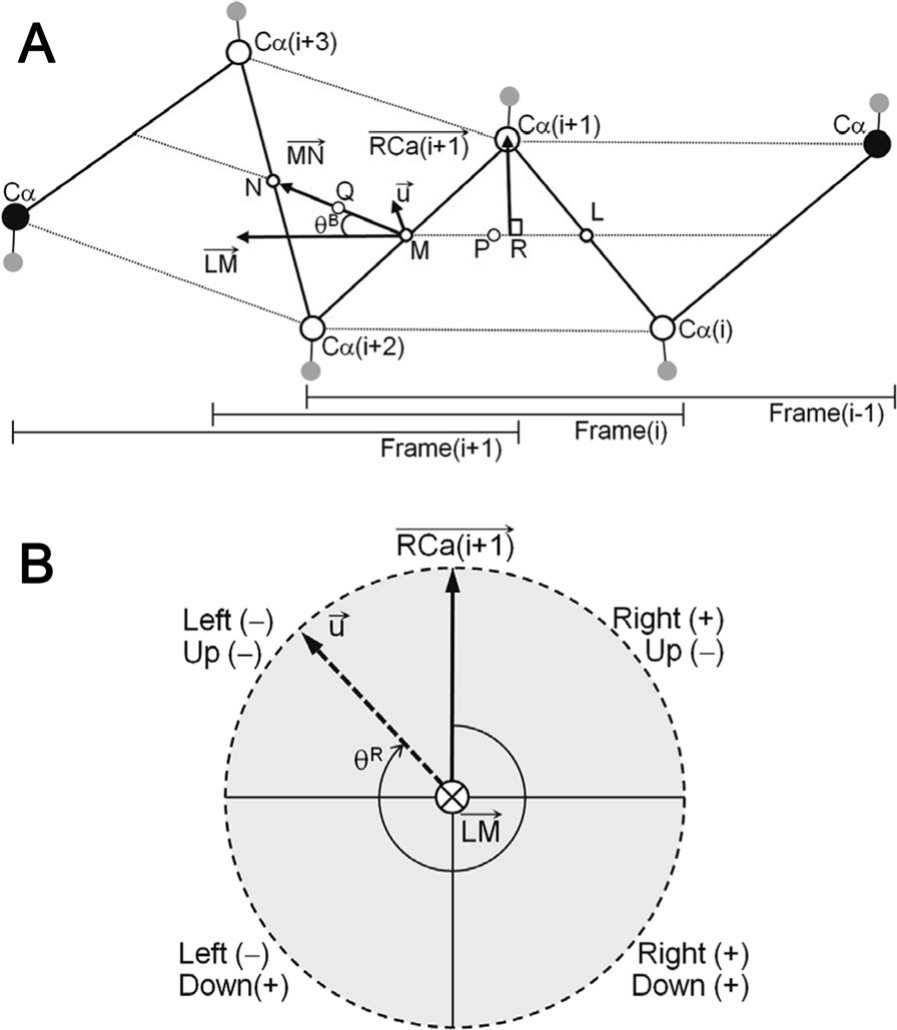
Definitions of twist and bend angles for a four-residue short β-strand frame. **a** Schematic of a six-α-carbon β-strand belt containing three frames. Open circles denoting Cα(i), Cα(i + 1), Cα(i + 2), and Cα(i + 3) represent the frame for which the twist and bend angles are calculated. The small gray circles represent the Cβ carbons. The letters L, M and N denote midpoints between two Cα carbons. The letters P and Q denote midpoints between L and M and between M and N, respectively. The twist angle is defined as the dihedral angle of Cα(i + 1), P, Q, and Cα(i + 2). The bend, θ^B^, is defined by the angle between the two vectors $\overrightarrow{LM}$ and $\overrightarrow{MN}$. The point R is the point at which the vector pointing from line $\overrightarrow{LM}$ to Cα(i + 1) is perpendicular. Vector $\overrightarrow{u}$ is the projection vector of $\overrightarrow{MN}$ on the plane that is perpendicular to $\overrightarrow{LM}$. Note that vector $\overrightarrow{u}$ is not on the plane containing Cα(i + 1), Cα(i + 2), and Cα(i + 3). (B) Schematic showing the possible signs of the bend angle. The two signs for the RL and UD directions are defined by the quadrant in which vector $\overrightarrow{u}$ resides. Vector $\overrightarrow{LM}$ points downward and is perpendicular to the plane of the dashed circle. The rotation angle, θ^R^, (0° < θ^R^ < 360°) is defined by the angle between the perpendicular vector $\overrightarrow{RC\alpha \left(i+1\right)}$ and the projection vector $\overrightarrow{u}$ of $\overrightarrow{MN}$ on the plane of the circle

For a short frame (defined by four consecutive Cα atoms), the midpoint between Cα(i) and Cα(i + 1) is defined as point L, the midpoint between Cα(i + 1) and Cα(i + 2) is defined as point M, the midpoint between Cα(i + 2) and Cα(i + 3) is defined as point N, and vector $\overrightarrow{RC\alpha \left(i+1\right)}$ is perpendicular to vector $\overrightarrow{LM}$ (Fig. 2a). Then, the midpoint between points L and M is defined as point P, and the midpoint between points M and N is defined as point Q. The twist angle, θ^T^, was defined as the dihedral angle of Cα(i + 1), P, Q and Cα(i + 2). The twist angle in a short frame of the trans state is 0° and ranges from–180° to 180°. The bend, θ^B^, in a short frame is defined by the angle between the two vectors $\overrightarrow{LM}$ and $\overrightarrow{MN}$. Then, we represented the direction of vector $\overrightarrow{MN}$ as the rotation angle, θ^R^, in a short frame (0° < θ^R^ < 360°) defined by the angle between the perpendicular vector $\overrightarrow{RC\alpha \left(i+1\right)}$ and the projection vector $\overrightarrow{u}$ of $\overrightarrow{MN}$ on the plane that is perpendicular to vector $\overrightarrow{LM}$, shown as the dashed circle in Fig. 2b. Each local bend angle is defined by a set of two signs (Fig. 2b). For the up or down (UD) direction, U is denoted by a negative sign (0° < θ^R^ < 90°, 270° < θ^R^ < 360°), and D is denoted by a positive sign (90° < θ^R^ < 270°). For the right or left (RL) direction, R is denoted by a positive sign (0° < θ^R^ < 180°), and L is denoted by a negative sign (180° < θ^R^ < 360°).

When $\overrightarrow{LM}$ and $\overrightarrow{MN}$ have the same direction, the bend angle is 0°. When $\overrightarrow{u}$ points to the left side of the circle (Fig. 2b), the RL sign is negative. Conversely, the RL sign is positive when $\overrightarrow{u}$ points to the right side of the circle. When $\overrightarrow{u}$ lies in the top half of the circle, the UD sign is negative. The UD sign is positive when $\overrightarrow{u}$ lies in the bottom half of the circle.

The distributions of local bend angles are normalized by the following equation to account for N', the number of times each angle is found.

2 $$ \mathrm{N}\hbox{'}=\frac{\mathrm{N}}{ \sin \left(\left|\uptheta \right|\right)} $$

where N is the number of frames in which the component of the bend angle is denoted θ [13]. Note that N is proportional to the circumference of a circle of radius sin|θ|. The calculation for a twist angle between Cα(i + 1) and Cα(i + 2) in a short frame has been described [13].

### β-Strand definition

DSSP (http://swift.cmbi.ru.nl/gv/dssp/) was used for secondary structure assignment. DSSP assigns secondary structures as H, α-helix; G, 3_10_-helix; I, 5-residue helix (π-helix); E, extended strand; B, residue in a β-bridge; S, bend; and T, hydrogen-bonded turn. For our study, we considered members of group E to be β-strands [15].

### Selecting protein structures to be included in the dataset

Non-redundant Research Collaboratory for Structural Bioinformatics Protein Data Bank (PDB: http://www.rcsb.org/pdb/) entries were prepared as described [16]. To facilitate the analysis, we extracted monomeric or homo-oligomeric and single-domain proteins from PDB. This has been accomplished previously with OLIGAMI (http://protein.t.soka.ac.jp/oligami/) [17], which is a database that combines the SCOPe database [18] with information pertaining to protein oligomerization [16] From these coordinates, we created a non-redundant set of PDB entries in which no pair of structures had >60 % sequence identity. Initially this set contained PDB data for analysis of fold dependence of secondary structure propensity on each amino acid. Therefore, the dataset included only SCOP folds that contained at least 2000 residues in β-strands. Consequently, we identified 24 (Additional file 1: 1874 PDB entries) SCOP folds for the dataset, with the number of frames included in our study being 47,435 [16]. Because there is a relationship between twist or bend angles and local amino acid composition [13, 16], we used this dataset to ensure consistency between the findings of our current study and those of previous studies. The residues in the buried region of β-strands were also identified in our previous study [16]. Amino acid residues were defined as “buried” when >80 % of the total accessible surface area was buried from solvent as described in detail in that previous study.

### Calculation of averaged UD and RL bend angles at each position in β-strands of the same length

To elucidate the relationship between the signs of the frame bend angles and their positions on a β-strand, we calculated the average bend angle at each frame position on β-strands of the same length after aligning their central frames. The β-strands were grouped by length with the members of each group aligned at the central frame. In the case of β-strands with an even number of frames, the central frame was defined as the frame immediately downstream of half the number of frames in the β-strand. For example, one strand has bend angles of alternating sign, and the sign of the central frame is positive. Another strand also has bend angles of alternating sign, but the sign of the central frame is negative. Simple averaging of bend angles at each position between these two strands will yield a small bend angle. To align these two strands before averaging the bend angles, if the sign of the local bend angle of the central frame on a given β-strand was negative, all local bend angles of its β-strand were multiplied by–1. Then, we could calculate the mean of the absolute values for the center residues. The average RL and UD bend angles for each position were calculated for each β-strand of a given length.

### Definitions of twist and bend angles for three-frame β-strand fragments

Fixed-length fragments consisting of six residues found in three consecutive frames were used to evaluate the twist and bend angles of full-length β-strands. The twist angle for each fragment was defined as the average angle of the three consecutive frames. The bend angles for the three-frame fragments were defined according to equation 3.

3 $$ {\uptheta}^{{\mathrm{UD}}_3}=\frac{\left|{\uptheta_{\mathrm{i}}}^{\mathrm{UD}}\hbox{-} {\uptheta}_{\mathrm{i}+2}^{{}^{{\mathrm{UD}}_3}}+{\uptheta}_{\mathrm{i}+2}^{{}^{\mathrm{UD}}}\right|}{3}, $$

where ${\uptheta}^{{\mathrm{UD}}_3}$ is the UD bend angles of the β-strand fragments.

### Identification of hydrophobic clusters

To identify and classify hydrophobic clusters, we used CluD [19] (http://mouse.belozersky.msu.ru/). This program considers each side-chain carbon or sulfur atom along with its covalently attached hydrogen atoms as a hydrophobic unit. We used the “strict atom list” of the program, where a carbon atom is no longer considered hydrophobic if it is bound to a hydrophilic group. We considered hydrophobic groups within 4.5 Å of each other as interacting. Other researchers have used the same cutoff distance to identify interacting hydrophobic groups [20, 21].

## Results and discussion

### Distribution of local bend angles

**Fig. 3:**
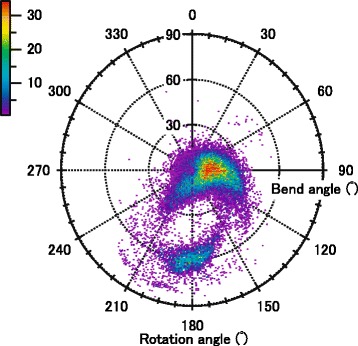
Relationship between the local bend and rotation angles of individual frames. The number of times a bend-rotation angle pair was found is represented by the color scale from purple to red (0–34 times)

The local β-strand bend and rotation angles were calculated separately for 47,639 short frames from 1867 PDB protein entries. Figure 3 shows the results in polar coordinates. Two clusters of bend/rotation angles are apparent. The main cluster (41,977 frames) is closer to the center of the circle with bend angles of <30°, with the majority of its frames (80.2 %, 33,648 frames) bent to the right, and with 48.5 % of its frames having rotation angles from 60 to 120°, a finding previously reported [13]. However, these observations do not mean that full-length β-strands will have right-hand bends because the perpendicular vector $\overrightarrow{RC\alpha \left(i+1\right)}$ of the neighboring frame will be oriented in the opposite direction, i.e., opposite sign, of the bend angle. If all frames in a β-strand have, for example, a 5° bend angle, the β-strand is not bent. The numbers of frames in the UD direction in the right half of this cluster are not significantly different (14,288 and 19,360 frames), but this is not the case for the RL direction, indicating that frames have UD bends causing full-length β-strands to bend in the UD direction as described in detail below.

There is also a cluster of short frames with bend angles and rotation angles of around 60° and 180°, respectively. As previously reported, when a frame has a large bend, it also has a large twist. The average of the absolute twist angles for frames in our dataset with bend angles >50° is 150° ± 24°, indicating that the side chains of the i + 1 and i + 2 residues are oriented in approximately the same direction (see Ref. 13 for the definition of the twist angle). Many of these frames, therefore, seem to be involved in a β-bulge, which is defined as a region between two consecutive β-type hydrogen bonds formed by two residues on one strand and one residue on an adjacent strand [22–24].

### Comparison of amino acid composition and angles

To investigate the relationship between the amino acid composition and the UD bends of the frames, the frames of the main cluster were divided into three groups according to their bend and rotation angles: large up bend (LU; 15° < θ^B^ < 30°, 0° < θ^R^ < 85°, 275° < θ^R^ < 360°), large down bend (LD; 15° < θ^B^ < 30°, 95° < θ^R^ < 265°), and small bend (S; θ^B^ < 4°). The frames with boundary rotation angles for UD directions (θ^R^; 90° ± 5°, 270 ± 5°) were not included in the LU and LD groups. The amino acid compositions of these groups and the bulge group (BU; θ^B^ > 50°, 150° < θ^R^ < 210°) were characterized and then compared (Table 1).

**Table 1:** Amino acid composition and propensities for each group

Amino acid	LU			LD			BU		S		P^β1a^	P^β2b^
	3694 frames			6804 frames			2659 frames		3381 frames			
	f^LU^(%)	f^LU^/f^S^	P^LU^	f^LD^ (%)	f^LD^/f^S^	P^LD^	f^BU^(%)	P^BU^	f^S^(%)	P^S^		
Val	14.6	1.1	2.21	14.1	1.1	2.13	13.7	2.07	13.0	1.97	2.00	1.87
Leu	12.4	1.3	1.38	10.4	1.1	1.15	10.1	1.12	9.4	1.05	1.15	1.22
Ile	10.7	1.2	2.05	9.7	1.1	1.86	10.1	1.93	8.9	1.71	1.79	1.67
Ala	7.7	1.1	0.93	6.2	0.9	0.75	7.5	0.90	6.8	0.82	0.75	0.72
Phe	6.4	1.0	1.64	6.2	0.9	1.59	4.2	1.07	6.6	1.69	1.40	1.33
Thr	5.2	0.7	0.90	6.3	0.9	1.09	5.1	0.88	7.3	1.26	1.21	1.17
Tyr	5.1	1.1	1.61	4.8	1.0	1.51	3.5	1.10	4.8	1.49	1.37	1.45
Gly	4.9	1.0	0.68	6.9	1.4	0.96	7.5	1.05	4.8	0.67	0.67	0.58
Ser	4.3	0.7	0.63	4.6	0.7	0.66	4.8	0.70	6.5	0.94	0.81	0.96
Glu	4.0	0.8	0.64	4.4	0.9	0.71	4.9	0.78	5.2	0.83	0.65	0.52
Lys	3.9	0.9	0.68	4.0	0.9	0.69	4.6	0.81	4.4	0.76	0.76	0.69
Arg	3.6	0.8	0.64	4.0	0.9	0.71	3.9	0.68	4.6	0.80	0.85	0.84
Asp	3.0	1.2	0.57	2.6	1.0	0.50	4.7	0.88	2.5	0.48	0.55	0.39
Asn	2.8	1.2	0.64	2.9	1.2	0.65	3.5	0.78	2.4	0.55	0.63	0.48
Gln	2.3	0.8	0.58	2.6	0.9	0.65	2.4	0.60	3.1	0.76	0.72	0.98
Met	2.2	1.1	0.91	1.9	1.0	0.78	1.5	0.63	1.9	0.81	1.01	1.14
His	2.1	0.8	0.96	2.1	0.8	0.97	2.4	1.08	2.5	1.14	0.99	0.80
Trp	1.7	0.9	1.32	1.8	1.0	1.41	1.4	1.10	1.8	1.39	1.23	1.35
Cys	1.7	1.0	0.98	2.0	1.2	1.18	1.6	0.96	1.7	1.00	1.36	1.40
Pro	0.9	0.7	0.17	2.0	1.6	0.39	2.2	0.43	1.3	0.25	0.40	0.31

^a^P^β1^, β-Sheet propensities reported by Fujiwara et al. [25]

^b^P^β2^, β-Sheet propensities reported by Williams et al. [25]

The propensities for the LU, LD, and S groups show similar tendencies for β-strands as reported previously [13, 25]. The LU and LD groups have very similar propensities and frequencies, indicating that the UD bend directions are not determined by the amino acid composition in a frame. The LU and LD groups have greater frequencies for the aliphatic residues Val, Ile, and Leu than do those in the S group, resulting in f^LU^/f^S^ and f^LD^/f^S^ >1.1 and suggesting that these residues are involved in large bending of a frame to the right as previously reported [13]. Interestingly, the f^LU^/f^S^ and f^LD^/f^S^ ratios of the aromatic residues are around 1.0. The f^LU^/f^S^ and f^LD^/f^S^ ratios are <0.9 for residues with polar or charged atoms in their side chains.

The BU frames often contain a Gly (7.5 %), especially at the i + 2 position (17.3 %), which is the greatest frequency found for the 20 amino acids at this position. As previously reported, Gly, because it lacks a β-carbon, cannot suppress a left-hand twist [9, 11]. Notably, frames that have left-hand twist angles have a left bend [13]. Therefore, not surprisingly, Gly appears frequently in the BU group. In the i + 2 position of the BU group, the frequencies of Asp, Asn, Glu, and Lys are also relatively large, and the frequencies of Phe and the β-branched residues Val, Ile, and Thr are substantially smaller, as previously reported [23].

The aforementioned results suggest that the local sequences in β-strands are related to their conformation and show that aliphatic residues are involved in large bend angles. However, knowledge of the amino acid sequence in a frame cannot be used to determine if the bend angle is LU or LD.

### Relationship between the local aliphatic-residue pattern and the bend angle in short frames

To examine how aliphatic residues in a short frame influence its bend and whether local aliphatic-residue patterns can discriminate between the U and D bend directions, we classified frames according to four aliphatic-residue patterns. We assigned Val, Leu, and Ile as aliphatic residues. When both the i and i + 2 residues with side chains pointing down in the frame were aliphatic, the frame was classified as AP1dw. When both the i + 1 and i + 3 residues with side chains pointing up in the frame were aliphatic, the frame was classified as AP1up. If all four residues in a frame were aliphatic, the frame was classified as AP2. All other frames were classified as AP0.

**Fig. 4:**
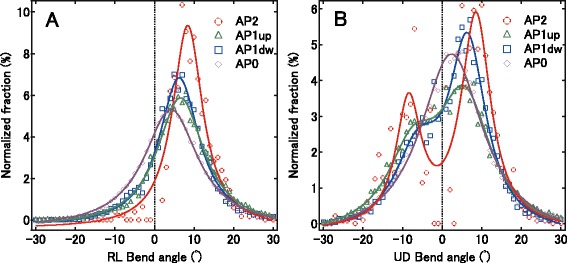
Relationships between distributions of short-frame bend angles and aliphatic-residue pattern. Normalized distributions for the RL and UD bends of the short frames according to their aliphatic-residue pattern classification. **a** The distributions for the RL bend angles were each fit with a Cauchy distribution, y = A/((x ‐ θ)^2^ + B) + y_0_. The peak angles for AP2, AP1up, AP1dw, and AP0 are 8.4 ± 0.2°, 6.6 ± 0.1°, 6.2 ± 0.1°, and 4.2 ± 0.1°, respectively. **b** The distributions for the UD bend angles were fit with double Cauchy distributions, y = A_1_/((x ‐ θ_1_)^2^ + B_1_) + A_2_/((x ‐ θ_2_)^2^ + B_2_)y_0_, except for the AP0 distribution, which was fit with a single Cauchy distribution. The peak angles (θ_1_ and θ_2_) for AP2 are −8.5 ± 0.6° and 8.5 ± 0.4°. The peak angles for AP1up are–7.4 ± 0.5° and 5.7 ± 0.3°. The peak angles for AP1dw are–6.1 ± 0.7° and 6.4 ± 0.2°. The peak angle for AP0 is 2.4 ± 0.1°

Figure 4a shows the RL bend angle distributions for AP2, AP1dw, AP1up, and AP0 (θ^B^ < 30°). All distributions display a single peak. The AP2 distribution, for which the frames contain the most aliphatic residues, has its peak at the largest angle, as previously reported [13]. The AP1up and AP1dw distributions are similar, indicating that the positions of the aliphatic-residue pairs are not important for the magnitude of the RL bend, although this conclusion does not apply to the number of aliphatic residues in a frame. The AP0 distribution has its peak at the smallest angle.

Conversely, with the exception of the UD bend angle distribution for AP0, the UD bend angle distributions for the other three groups are bimodal regardless of the local aliphatic residue pattern (Fig. 4b). It is clear that a local interaction involving aliphatic residues i.e., a hydrophobic interaction, is not a determinant for the sign of the UD bend angle because local pairs of aliphatic residues residing on the same side of a β-strand allow for a bimodal distribution of UD bend angles. Therefore, the determinant for the sign of a UD bend angle probably is dependent on long-range interactions.

### Relationship between aliphatic-residue content and frame length

The distributions of the RL bend angles have a single positive peak, i.e., a right bend angle, indicating that the Cα atoms in β-strands zigzag and suggesting that β-strands are shorter than an extended polypeptide chain, a well-known and intuitive conclusion. Because the bend angle values increase with an increasing number of aliphatic-residue pairs as described above, a frame with an aliphatic-residue pair should be shorter than a frame without one. To examine the relationships between the length and bend of the frames, for frames that have small (θ^B^ < 4°) or large (15° < θ^B^ < 30°) bend angles, the average length between Cα(i) and Cα(i + 3) in frames with eight inter-strand hydrogen bonds was calculated for the four groups. As expected, the average frame lengths with larger bend angles are shorter than those with smaller bend angles for all four groups (Table 2). Furthermore, the AP2, AP1dw, and AP1up frame lengths are shorter than those of AP0 frames, which do not have at least one set of aliphatic side chains on the same side of the β-strand in close contact. Consequently, contraction of β-strands is apparently controlled by hydrophobic interactions within a frame such that the local bending of the frame is enhanced.

**Table 2:** Average distances between Cα(i) and Cα(i + 3) in local frames

	Small bend (θ^B^ < 4°)			Large right bend (15° < θ^B^ < 30°)		
Group	No. of frames	Average distance	Average angle	No. of frames	Average distance	Average angle
AP2	5	10.10 ± 0.05	3.0 ± 0.6	22	9.66 ± 0.05	17.8 ± 0.6
AP1dw	107	10.12 ± 0.02	1.8 ± 0.2	167	9.72 ± 0.02	19.4 ± 0.3
AP1up	56	10.20 ± 0.03	2.0 ± 0.3	222	9.74 ± 0.02	18.4 ± 0.2
AP0	401	10.27 ± 0.01	1.6 ± 0.1	900	9.85 ± 0.01	19.4 ± 0.1

### Bending direction for a full-length β-strand

A full-length β-strand does not have a large bend if the bend angles of consecutive frames have the same sign, but it is bent when its adjacent frames have bend angles of alternating signs. The distributions of the RL bend angles for the four groups peak unimodally at relatively small positive values (Fig. 4a), indicating that full-length strands should be approximately straight. For the four aliphatic–residue patterns, the distributions of their UD bends are bimodal except for those of AP0, suggesting that the β-strand frames for AP2, AP1dw, and AP1up have alternating signs and therefore induce bent β-strands (Fig. 4b).

**Fig. 5:**
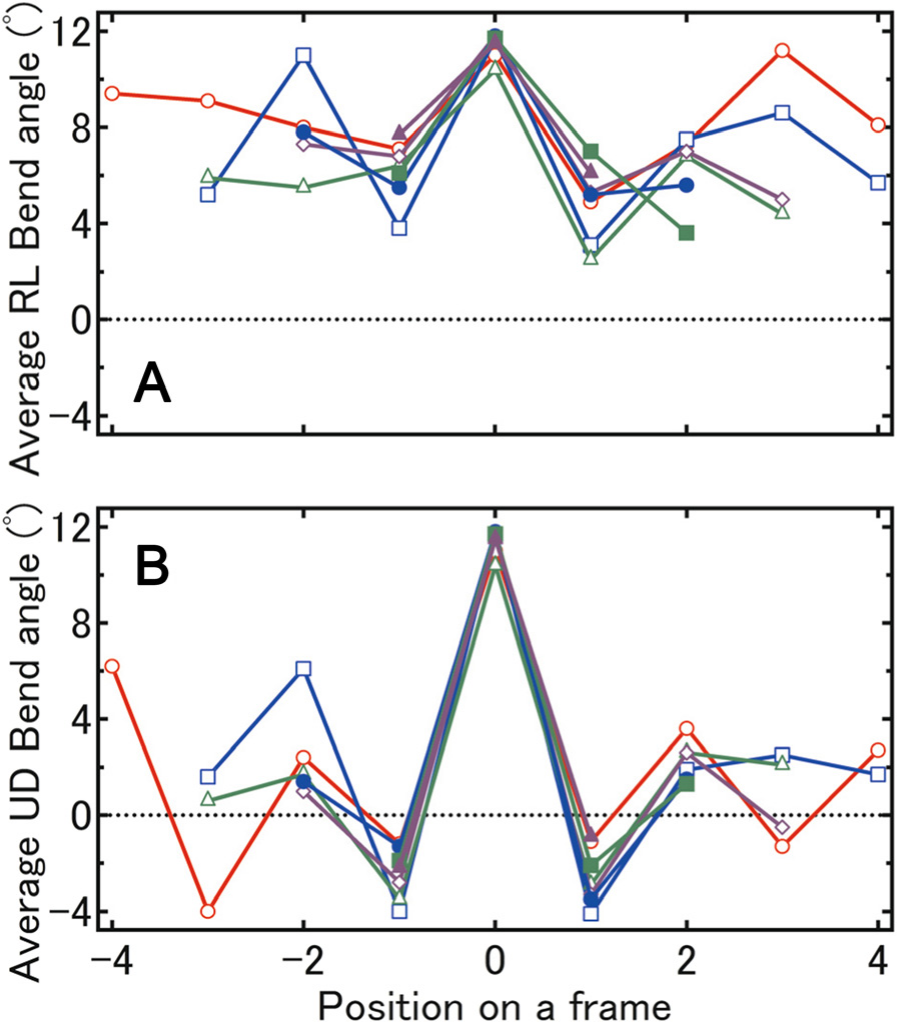
Average bend angle at each position on a β-strand fragment. Average bend angle at each position on a β-strand fragment for the (**a**) RL and (**b**) UD bend angles. The lengths of the β-strands, with three, four, five, six, seven, eight, or nine frames, are represented by the colors, green, purple, orange, cyan, magenta, blue and red, respectively. Symbols (triangle or square) represent β-strands with an odd or even number of frames

To elucidate the relationship between the signs of the frame bend angles and their positions on a β-strand, we calculated the average bend angles at each frame position on β-strands of the same length after aligning their central frames. As shown in Fig. 5a, the average RL bend angles for all positions are positive, indicating that full-length β-strands do not have a large bend induced by the RL local bends. Conversely, as shown in Fig. 5b, the signs of the UD bend angles for the individual positions alternate between negative and positive values, indicating that the strands should be bent in the UD direction of each β-strand.

The average UD and RL angle values of the central frames are more positive than are those of the other frames because the angles of all central frames were assigned positive values (see Methods). Conversely, the averaged values for the other positions can be negative or positive. Straight and bent strands are found for the AP2 group. As noted above, consecutive frames have UD bend angles of alternating sign in bent strands, whereas straight strands contain frames with only positive bend angles. Therefore, the peak for frames with positive bend angles will be higher than that for negative bend angles (Fig. 4b).

### Relationship between the twists and bends of full-length β-strands

The alternating signs of the UD bend angles for consecutive frames produce the large bends found for full-length β-strands. We defined ${\uptheta}^{{\mathrm{UD}}_3},$ the bend angle with the three consecutive frames containing six residues. The frames of the Rheb strands B and C (Fig. 1b) have alternating signs, and consequently the strands have large bends (Table 3), causing them to coil around the D-helix. The values of all three-frame fragments of the C strand are ≥12.9°. For the B strand, the last two three-frame fragments have large ${\uptheta}^{{\mathrm{UD}}_3}$ values (12.0°, 16.2°). The N-terminus of the A strand has a gentle curve, and the ${\uptheta}^{{\mathrm{UD}}_3}$ value of the first three-frame fragment is 10.0°. Conversely, strands D and E are almost straight and have only small ${\uptheta}^{{\mathrm{UD}}_3}$ values (D: 3.9° and 2.2°; E: 3.6°). Table 3 also shows the average twist angles, ${\uptheta}^{{\mathrm{UD}}_3},$ of the three-frame fragments.

**Table 3:** Twist and bend angles of the local frames and the three-frame β-strand fragments in Rheb

Strand	Frame No.	Residue No.	θ^Ta^	θ^UDb^	θ^T^_3_^c^	θ^UD^_3_^d^
A	1	5	–4.1	6.4	12.0	10.0
	2	6	32.2	–18.0	21.4	3.3
	3	7	7.9	5.6	19.1	2.7
	4	8	24.2	13.5	16.3	2.8
	5	9	25.3	16.0		
	6	10	–0.5	11.0		
B	1	41	15.6	10.1	16.1	0.3
	2	42	35.0	18.8	19.5	1.6
	3	43	–2.4	7.8	2.1	12.0
	4	44	26.0	–16.0	14.3	16.2
	5	45	–17.3	12.3		
	6	46	34.2	–20.3		
C	1	52	15.8	10.5	17.2	14.8
	2	53	21.5	–18.5	16.0	15.8
	3	54	14.3	15.3	13.8	13.1
	4	55	12.3	–13.5	15.7	12.9
	5	56	14.7	10.6		
	6	57	20.2	–14.6		
D	1	80	19.4	–14.6	13.4	3.9
	2	81	13.3	9.0	4.2	2.2
	3	82	7.7	11.8		
	4	83	–8.2	–3.9		
E	1	114	25.0	15.0	18.0	3.6
	2	115	10.9	9.0		
	3	116	18.0	–16.9		
F	1	144	–26.0	10.5		

^a^θ^T^, twist angle of the frame

^b^θ^UD^, bend angle of the frame

^c^θ^T^_3_, twist angle of the three-frame β-strand fragment

^d^θ^UD^_3_, bend angle of the three-frame β-strand fragment

**Fig. 6:**
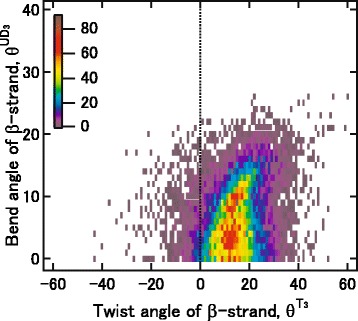
Relationships between the twist and bend angles of the three-frame fragments. The number of times a bend-twist angle pair was found is represented by the color scale from purple to red (0–88 times) shown in the figure

Figure 6 shows the relationship between the ${\uptheta}^{{\mathrm{T}}_3}$ and ${\uptheta}^{{\mathrm{UD}}_3}$ values for 14,877 of the fragments surveyed, none of which have abnormally large bends (θ^B^ < 30°). The majority of the ${\uptheta}^{{\mathrm{T}}_3}$ values are positive, and the most frequent value is 13°, which is the same as the most frequent θ^T^ value reported previously [13]. There are two peaks at ${\uptheta}^{{\mathrm{UD}}_3},$ around 4° and 9°, which are similar to the peak angles of AP0 and AP2, respectively. As seen in Fig. 6, the ${\uptheta}^{{\mathrm{UD}}_3}$ values are not related to the ${\uptheta}^{{\mathrm{T}}_3}$ values (correlation coefficient = 0.25), although the local bend angles correlate with the local twist angles [13]. These results indicate that the bending of a full-length β-strand is independent of its twist, which is different from the relationship between the short-frame bend and twist angles.

### Origin(s) of bends in full-length β-strands

We reported that the local RL bend angle correlates with the local twist angle in a given short frame [13]. Furthermore, the local RL bend angle correlates with the number of aliphatic residues in a frame in a manner different from that of the twist angle, which correlates negatively with the number of hydrophilic, but not hydrophobic, residues [13]. For this study, we found that β-strand contraction is a consequence of hydrophobic interactions within at least one frame, which enhances the local bending of the frame. This leaves us with the question: what causes the bending of a full-length β-strand?

**Fig. 7:**
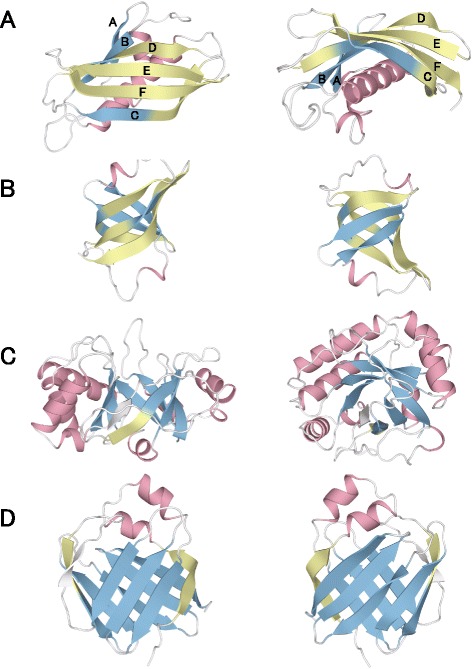
β**-**strand conformations for four different protein folds. Ribbon diagrams of the (**a**) thioesterase/thiol ester dehydrase-isomerase, (**b**) OB, (**c**) TIM β/α barrel, and (**d**) lipocalin folds (PDB ID: 1SC0, 1LM0, 1SFS, and 1KQW, respectively). The β-strand regions that are bent and straight are colored green and blue, respectively. Because the β-strands colored white have only one frame, θ^UD2^ could not be calculated. The α-helices are colored pink

Rheb strands B and C have large bends and pack against the surface of helix D, which has its long axis perpendicular to those of the β-strands. A similar interaction is found for the thioesterase/thiol ester dehydrase-isomerase fold (Fig. 7a). The four β-strands in this fold bend over an α-helix. The five β-strands of the oligonucleotide/oligosaccharide binding fold—an example of a fold containing only β-strands—form a small barrel structure with a small hydrophobic core (Fig. 7b), and the three longer β-strands of the fold roll around this small hydrophobic core.

**Fig. 8:**
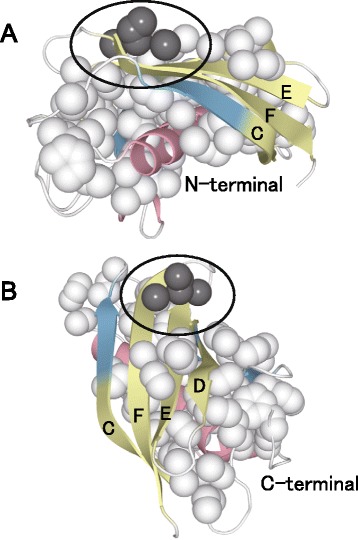
Hydrophobic clusters in the thioesterase/thiol ester dehydrase-isomerase fold. Hydrophobic clusters in thioesterase/thiol ester dehydrase-isomerase (PDB ID: 1SC0) viewed from two orientations. The N-terminus of the α-helix is visible in (**a**), and its C-terminus is visible in (**b**). The spheres denote the hydrophobic carbon or sulfur atom clusters as identified by Clud (http://mouse.belozersky.msu.ru/). The hydrophobic strand F atoms colored dark gray may induce the bend in strand F

Conversely, the Rheb strands D and E do not have large bends. The common feature of these two β-strands is that the α-helices that abut them are oriented parallel to the β-strands. A second example of a fold with parallel helices and β-strands is the TIM β/α barrel fold. This fold has six straight β-strands surrounded by α-helices oriented in parallel, and, notably, the only β-strand that does not interact with an α-helix has a large bend (Fig. 7c). The lipocalin fold has two large β-sheets that overlap and do not contact the two α-helices (Fig. 7d). Except for the two edge β-strands, the β-strands of the lipocalin fold are not bent. The β-strands of one β-sheet cross three or four β-strands of the other β-sheet, with a wide, flat hydrophobic area between the two sheets. Notably, the hydrophobic cluster around the C-terminal region of β-strand F in the thioesterase/thiol ester dehydrase-isomerase fold is not large enough to cause it to bend (Fig. 8). Because the surface of the N-terminal region of the α-helix that interacts with β-strand F is populated with only Gly and Ser side chains, the C-terminal region of β-strand F, which contains Leu, Val, and Arg residues, does not have a hydrophobic interaction partner. Instead, the hydrophobic cluster of the C-terminal region of β-strand F folds back on itself in an upward direction. These observations suggest that β-strands bend to maintain hydrophobic interactions with the abutting hydrophobic surface. However, when a hydrophobic surface can completely or almost completely abut the long axis of a β-strand, the β-strand is expected to be almost straight. β-strands are more flexible than are α-helices and can bend perpendicular to their long axes to interact with an abutting hydrophobic surface, or they can bend to form a hydrophobic side-chain cluster when an abutting hydrophobic surface is absent.

**Fig. 9:**
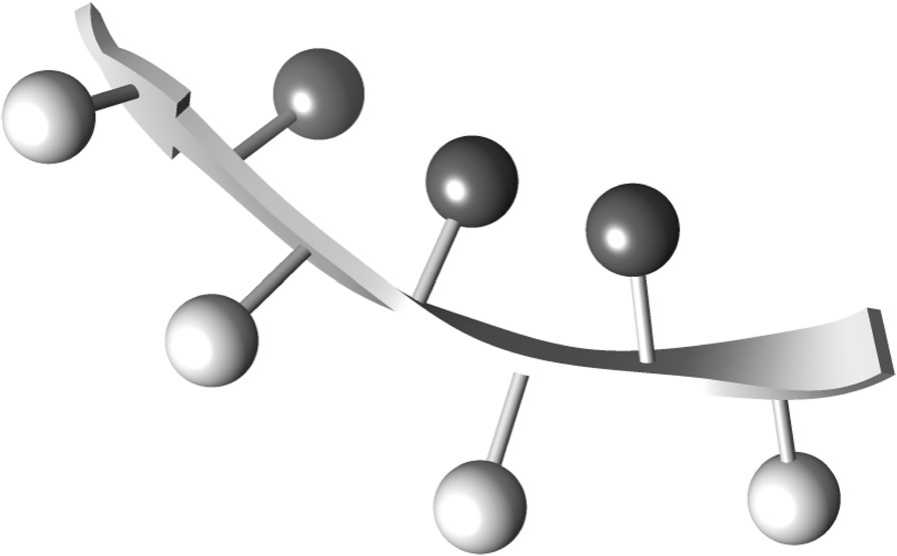
Schematic of the condensed and extended sides in β-strands with large bends. The dark- and light-gray spheres represent the side chains of the condensed and extended sides in β-strands with large bends, respectively

We compared the amino acid propensities on the two sides of the large bend fragments (9 < ${\uptheta}^{{\mathrm{UD}}_3}$ < 19°) with the condensed side (CS) represented by dark-gray spheres and the extended side (ES) represented by light-gray spheres (Fig. 9). The amino acid propensities for both sides of small bend fragments (SF; ${\uptheta}^{{\mathrm{UD}}_3}$ < 4°) were also calculated. As shown in Table 4, large values for the propensities, P^CS^, P^SF,^, and P^ES^ were obtained for the hydrophobic and aromatic residue. Additionally, Val, Tyr, Phe, and Trp are preferred at CS than ES, resulting in P^CS^/P^ES^ values >1.1. Leu and Ile have large P^CS^ and P^ES^ values, but these values are approximately equal, indicating that Leu and Ile are equally preferred at CS and ES and, therefore, do not contribute to the bending of a full-length β-strand. These results show that the driving force for full-length β-strand bending is different from that for the local bending. Using the knob-socket model, Joo and Tsai showed that aromatic residues are favored for tertiary packing in structures such as inter-β-sheets, whereas aliphatic residues are frequently involved in intra-β-sheet interactions between the i and i + 2 residues of a strand or as inter-strand residues forming backbone hydrogen bonds [26]. In fact, 58 proteins of the OB fold (Fig. 7b), which has highly bent β-strands, have a remarkably high content of the aromatic residues Tyr, Phe, and Trp (21.8 %) in buried regions of β-strands, although the TIM β/α barrel fold (261 proteins) and lipocalin fold (41 proteins) (Fig. 7c and d), which have slightly bent β-strands, have a low content of aromatic residues (12.4 % and 10.8 %, respectively) in buried regions of β-strands. In particular, it is interesting that both the OB and TIM β/α barrel folds have large local twist and bend angles as shown in our previous study [13]. These findings also support the conclusion that the bending of a full-length β-strand is caused by tertiary interactions of aromatic residues. Furthermore, both the OB and lipocalin folds have antiparallel β-sheets, indicating that structural features such as parallel or antiparallel do not influence the bending of full-length β-strands.

**Table 4:** The amino acid propensities for β-strands with large bends

Amino acid	P^CS^	P^SF^	P^ES^
Val	2.15	2.03	1.76
Ile	1.91	1.84	1.85
Tyr	1.74	1.71	1.51
Phe	1.73	1.63	1.58
Trp	1.56	1.28	1.41
Leu	1.24	1.09	1.25
Cys	1.19	1.19	1.21
His	1.11	1.07	1.13
Thr	1.01	1.23	1.19
Met	0.91	0.78	0.80
Ala	0.90	0.75	0.65
Glu	0.71	0.75	0.86
Ser	0.71	0.81	0.75
Gly	0.67	0.67	0.68
Gln	0.66	0.74	0.71
Asn	0.63	0.67	0.63
Arg	0.62	0.79	0.90
Lys	0.61	0.83	0.93
Asp	0.46	0.51	0.62
Pro	0.27	0.25	0.23

### Robustness of the dataset

We checked the robustness of our results using a sub-dataset of SCOP folds that contained less than 2000 residues, which was not included in the dataset we used in our present study (1916 entries). The amino acid propensities P^CS^, P^SF^, P^ES^ from the sub-dataset were very similar to those listed in Table 4 in that the deviation was <0.2, except for P^ES^ values for Cys and Trp residues. P^ES^ values are 0.83 and 0.97 for Cys and Trp, respectively, in the case of the sub-dataset, which did not affect our conclusions. Therefore, our results seem to be independent of dataset selection.

## Conclusions

This work, for the first time, presents a detailed analysis of the bend angle of full-length β-strands in globular proteins with known three-dimensional structures. We conclude that the dominant force that drives the bending of a full-length β-strand in the UD direction is hydrophobic interactions involving aromatic residues, whereas that for local β-strand bends is hydrophobic interactions involving aliphatic residues. These findings will be applicable for the detailed design of β-strands, which have far more structural diversity than α-helices. For example, aromatic residues can be inserted into specific sites within a polypeptide to engineer a bent β-strand, and a straight strand can be engineered by substituting with aliphatic residues such that they can interact hydrophobically with a partner structure such as a long α-helix.

## Additional file

Additional file 1:
**List of PDB codes.** The file contains a list of all the PDB codes and protein names used in our study. (TXT 139 kb)

## Abbreviations

RL: Right-and-left
UD: Up-and-down
LU: Large up
LD: Large down
S: Small
BU: Bulge
CS: Condensed side
ES: Extended side
SF: Small bend fragment
